# Care-seeking patterns amongst suspected paediatric pneumonia deaths in rural Malawi

**DOI:** 10.12688/gatesopenres.13208.2

**Published:** 2021-05-06

**Authors:** Carina King, Masford Banda, Naor Bar-Zeev, James Beard, Neil French, Charles Makwenda, Eric D McCollum, Malizani Mdala, Yasir Bin Nisar, Tambosi Phiri, Shamim Ahmad Qazi, Tim Colbourn

**Affiliations:** 1 Department of Global Public Health, Karolinska Institute, Stockholm, Sweden; 2 Institute for Global Health, University College London, London, UK; 3 Parent and Child Health Initiative, Lilongwe, Malawi; 4 Centres for Disease Control and Prevention, Lilongwe, Malawi; 5 Department of International Health, Johns Hopkins Bloomberg School of Public Health, Baltimore, USA; 6 Institute of Infection, University of Liverpool, Liverpool, UK; 7 Department of Pediatrics, Johns Hopkins Medicine, Baltimore, USA; 8 Department of Maternal, Newborn, Child and Adolescent Health and Ageing, World Health Organization, Geneva, Switzerland

**Keywords:** mortality, pneumonia, child, sub-Saharan Africa, pathways to survival

## Abstract

**Background: **Pneumonia remains a leading cause of paediatric deaths. To understand contextual challenges in care pathways, we explored patterns in care-seeking amongst children who died of pneumonia in Malawi.

**Methods: **We conducted a mixed-methods analysis of verbal autopsies (VA) amongst deaths in children aged 1-59 months from 10/2011 to 06/2016 in Mchinji district, Malawi. Suspected pneumonia deaths were defined as: 1. caregiver reported cough and fast breathing in the 2-weeks prior to death; or, 2. the caregiver specifically stated the child died of pneumonia; or 3. cause of death assigned as ‘acute respiratory infection’ using InterVA-4. Data were extracted from free-text narratives based on domains in the ‘Pathways to Survival’ framework, and described using proportions. Qualitative analysis used a framework approach, with pre-specified themes.

**Results:** We analysed 171 suspected pneumonia deaths. In total, 86% of children were taken to a healthcare facility during their final illness episode, and 44% sought care more than once.  Of children who went to hospital (n=119), 70% were admitted, and 25% received oxygen. Half of the children died within a healthcare setting (43% hospital, 5% health centre and 2% private clinics), 64 (37%) at home, and 22 (13%) in transit. Challenges in delayed care, transport and quality of care (including oxygen), were reported.

**Conclusions:** Healthcare was frequently sought for children who died of suspected pneumonia, however several missed opportunities for care were seen. Sustained investment in timely appropriate care seeking, quick transportation to hospital and improved case management at all levels of the system is needed.

## Introduction

Annually approximately 800,000 children aged under five years die from pneumonia. While the absolute number of deaths declined by 65% between 1990 and 2017, pneumonia remains the leading cause of infectious paediatric deaths ^1^. Globally, pneumonia incidence is higher amongst male children, while mortality is higher amongst female children; however, this is not consistent across regions and the mechanisms for this trend are poorly understood ^2^. Addressing paediatric pneumonia deaths will be crucial to achieving Sustainable Development Goal 3.2 in an equitable way ^3^.

Wide-spread adoption of standardised integrated community case management (iCCM) and Integrated Management of Childhood Illnesses (IMCI) protocols, alongside routine childhood vaccination, have led to significant reductions in paediatric pneumonia mortality ^1, 4^. These approaches aim to screen children at community and primary care levels, and refer those with severe illnesses to higher levels of care. However, there is evidence of poor-quality implementation for pneumonia assessment and management ^5– 8^.

For these approaches to be optimised, caregivers at home must recognise signs of pneumonia, decide to seek, and be able to seek, care. A 2018 study estimated 60% of preventable deaths occur from poor-quality care, rather than issues in accessing care ^9^. However, there is conflicting evidence around whether care is sought in time or too late during acute childhood illnesses, including pneumonia, and considerable evidence gaps remain ^10, 11^.

Several frameworks have been developed to understand processes and barriers that contribute to mortality across the continuum from community to referral facility. The Three Delays model, developed for maternal mortality, breaks down challenges into delays in deciding to seek care, delays in reaching care, and delays in receiving appropriate care ^12^. More specifically for childhood illnesses, the Pathways to Survival model was developed to support IMCI implementation, and arranges steps for communities and healthcare systems to take to promote survival ^13^.

To prevent paediatric pneumonia deaths, context specific understanding of caregiver, community and health system barriers to quality care are needed. We aimed to describe caregiver recognition of illness, care-seeking decisions and quality of care issues amongst families with a child pneumonia death in Malawi, and explore whether these patterns differed by sex or age. 

## Methods

We conducted a mixed-methods analysis of verbal autopsy (VA) data from a prospective community-based birth cohort in Mchinji District, central Malawi, amongst deaths in children aged 1 to 59 months from October 2011 to June 2016 ^14^.

### Setting

At the time of data collection Mchinji district had an approximate population of 450,000, with 85% living as rural subsistence farmers. The under-five mortality rate was 63/1000 livebirths in the 2015–2016 Malawi Demographic and Health Survey. Healthcare was provided for free by 354 community healthcare workers (known locally as Health Surveillance Assistants), 11 government primary healthcare centres, and one referral district hospital, and for a small fee in four rural hospitals.

### Data collection

Full details of the community surveillance system have been published previously ^14^. Briefly, deaths were recorded and reported monthly by 1059 village-level key informants. Deaths were also identified at household visits conducted for all children born in the district at four months and one year of age. Data were submitted to the office for cleaning. A list of all community reported deaths amongst 0–59 months olds in Mchinji district was generated monthly for VA interviews. VAs were conducted by nine senior fieldworkers, all with previous experience of conducting VAs. They received one week’s training, including: translating the data collection tool, conducting mock interviews and using smart phones for data collection.

VAs interviews were randomised to conduct an open narrative or not, as part of a separate research question on the role of narratives in data quality, rapport and interview procedures ^15^. This was done at the point of interview using a random number generator within the Open Data Kit (ODK) Collect application, version 1.4, used to collect data ^16^. The open narrative was unstructured, conducted at the start of the VA and was either audio recorded then transcribed or captured using paper notes. Data collectors could record the information in the format they preferred, in English or Chichewa. Open narratives in Chichewa were later translated by the data collectors and data entry clerks. We used the standardized World Health Organization (WHO) 2012 VA questionnaire ^17^.

### Pneumonia death definition

Suspected pneumonia deaths were identified in three ways: 1. the caregiver responded yes to both the child having a cough and fast breathing in the two weeks prior to death, according to the WHO 2012 VA questionnaire; 2. the caregiver explicitly stated that the child had died of pneumonia during the VA; or 3. the cause of death was classified as ‘acute respiratory infection’ by InterVA-4. We excluded deaths occurring in the neonatal period (aged 0–28 days). 

### Quantitative analysis

We extracted data from the open narratives using a custom-designed tool in Excel, based on the ‘Pathways to Survival’ framework ( Table 1) ^13, 18^. We did not consider indicators relating to wellness inside the home (e.g. breastfeeding, hygiene) or outside the home (e.g. imminisation, water and sanitation), or indicators of quality of care inside the home. The tool was developed by TC and CK using three randomly selected narratives to define fields and categories. Data were then double coded from a random set of ten narratives. Disagreements between coding were discussed and the data extraction tool updated; a further 10 narratives were double coded to check for consistency. The remaining narratives were coded by CK. Data were described using proportions and means, and compared with chi ^2^ and t-tests. We stratified patterns of care-seeking by sex and age groups.

**Table 1.** List of indicator definitions extracted from open narratives.

Indicator	Definition	Categories
Recognised illness	The respondent mentions any clinical sign of infection, diagnosis or acute illness. We did not include witchcraft or curses.	Yes, No, Don’t know
Recognised pneumonia	The respondent mentions cough, difficulty or fast breathing, noisy breathing, chest indrawing, or states the child had pneumonia or an acute respiratory infection.	Yes, No, Don’t know
Time taken to first seek care *	The approximate time from first recognising the child was sick to seeking care outside of the home.	<24 hours 24–72 hours >72 hours Don’t know
Home care given	The respondent described providing any form of home-based treatment, such as medication or feeding, outside of healthcare advice (i.e. giving antibiotics at home as prescribed by a healthcare provider was not coded).	Yes, No, Don’t know
Location of care- seeking	The location where the respondent stated they went to seek care, if care was sought outside of the home.	None sought Traditional medicine Community health worker Health centre Hospital Private clinic Don’t know
Quality of care issue	There is mention of any aspect of poor quality of care, including: lack of staff, equipment, medication, transport, delays and negative staff attitude.	Text description
Action on care- seeking	The action that was taken or advised by a healthcare worker, while seeking care at a facility or provider. If a child was referred but never arrived at the facility, this was recorded as being referred.	Sent home Admitted Referred to another facility Don’t know
Total duration of illness	The approximate time from first recognising the child was sick to the child dying.	Number of days
Location of death	The location where the child died was explicitly stated or could be determined from the narrative (e.g. ‘after they died we were discharged’).	Home In transit Health centre Hospital Private clinic Don’t know
Oxygen given	The respondent stated that the child was given oxygen treatment, including terms such as ‘breathing tube’.	Yes No Don’t know
Other illness	The respondent described a long-term or chronic illness (e.g. asthma), malnutrition, a birth defect, or either being premature or low-birthweight.	Text description.

* We interpreted several as 3 days and couple 2 days

### Qualitative analysis

Narratives were analysed using a framework approach ^19^. Themes were pre-defined, based on the Pathway to Survival framework ^18^, as: wellness, illness recognition, care-seeking decisions, referral procedures, and quality of care. Concepts were inductively coded within these themes by CK, and a random sub-set of ten narratives were double coded by TC. The interpretation of the data was discussed with two of the data collectors who conducted VAs to check for context-appropriate interpretation. Disagreements in interpretation were discussed until consensus was reached.

### Ethics

The data used were collected as part of the VacSurv Study, which was approved by the National Health Sciences Research Ethics Committee in Malawi (reference: 837), London School of Hygiene and Tropical Medicine (reference: 6047) and Centers for Disease Control and Prevention (reference: 6268). Verbal informed consent was taken from all respondents, and confirmed in the electronic data form; written consent was not sought due to literacy rates.

### Patient and Public Involvement

Before starting any data collection, the study protocol was presented to the District Executive Committee and District Health Management teams in Mchinji for input and approval. Extensive community engagement was conducted through village development committees and traditional leaders. Community consent was sought from traditional authorities during study introduction, who also assisted in informing communities of verbal autopsies.

## Results

### Cohort description

**Figure 1.**
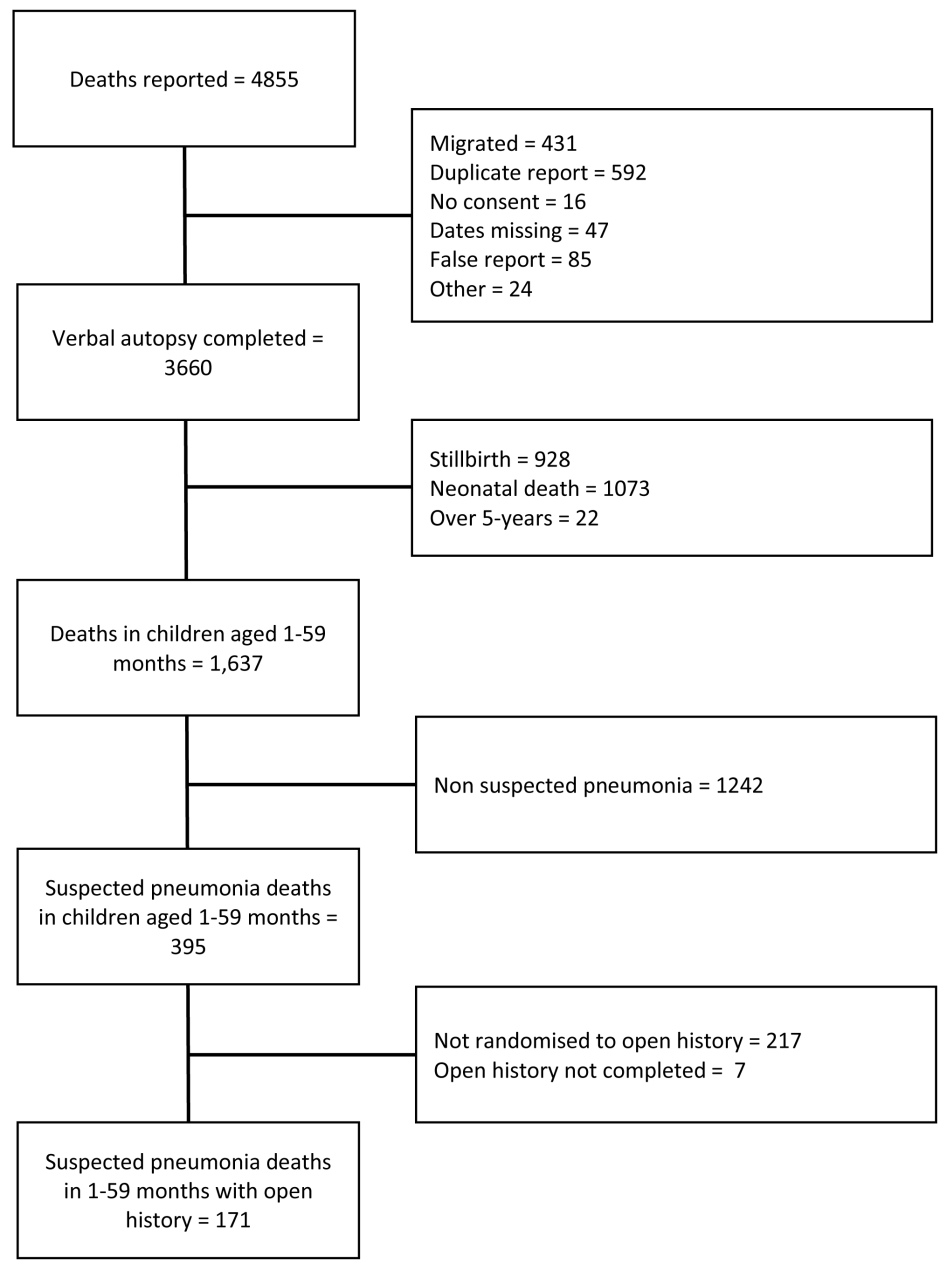
Participant inclusion flow-chart.

During the cohort period, 4,855 death events were reported ( Figure 1). Of the 1673 confirmed post-neonatal under-five deaths, 395 (24%) were classified as suspected pneumonia, representing a post-neonatal pneumonia mortality rate of 8.2 per 1000 livebirths. A total of 178 (45%) were randomised to open narrative, with balance between the two arms in terms of key demographics and care-seeking proxies ( Table 2). Overall 171 suspected pneumonia deaths with complete data were included in analysis. Of these 37% (n=64/171) were completed in Chichewa and translated to English. The majority of deaths were classified as suspected pneumonia by InterVA (153/171, 89%).

**Table 2.** Description of suspected-pneumonia deaths, according to narrative allocation.

		Open Narrative * N = 171	No Narrative N = 217
Variable		N (%)	N (%)
Age	1–11 months 12–59 months	124 (73%) 47 (27%)	151 (70%) 66 (30%)
Sex	Male Female Missing	74 (43%) 96 (56%) 1 (1%)	103 (47%) 113 (52%) 1 (<1%)
Sought formal care prior to death **	Yes No Don’t know	147 (86%) 23 (14%) 1 (<1%)	187 (86%) 29 (13%) 1 (<1%)
Location of death	Home Healthcare facility In transit Other *** Missing	64 (37%) 82 (48%) 22 (13%) 3 (2%) -	72 (33%) 97 (45%) 19 (9%) 18 (8%) 11 (5%)
Season of death	Rainy Dry	94 (55%) 77 (45%)	124 (57%) 93 (43%)
Pneumonia classification	InterVA only WHO VA signs only Caregiver reported only InterVA + WHO VA signs WHO VA signs + caregiver InterVA + caregiver InterVA + WHO VA signs + caregiver	106 (62%) 10 (6%) 7 (4%) 31 (18%) 1 (1%) 13 (8%) 3 (2%)	135 (62%) 21 (10%) 8 (4%) 36 (17%) 2 (1%) 10 (5%) 5 (2%)

*Only the open narrative records are included in the analysis **Question from the WHO 2012 VA tool – “In the final days before death, did (s)he travel to a hospital or health centre” ***’Other’ includes private clinics and outside of the household (e.g. with a neighbour or in the field).

### Quantitative analysis

Overall, 86% (n=146/171) of children were taken to a public or private healthcare facility during their final illness episode. 44% (n=76/171) of caregivers sought care more than once in the final illness episode, with the most reported attendances being six over a 35-day period, visiting a health centre twice and hospital four times ( Table 3). A sole visit to hospital occurred in (n = 50/171) 29%, and a single visit to a health centre in (n=17/171) 10%. Care-seeking at pharmacy shops was not explicitly reported. Of those where information on time to care-seeking could be extracted, 44% (n=36/81) of caregivers sought care within 24 hours of illness recognition. The median time from illness recognition to death was three days (IQR: 1 - 5). In 48 narratives (28%), the caregiver stated the child has a pre-existing condition or risk factor, such a birth defect or being born prematurely.

**Table 3.** Description of location of care-seeking.

	First (n = 162) *	Second (n = 76)	Third (n=29)	Fourth (n=10)	Fifth (n=4)	Sixth (n=1)
No care sought	14 (9%)					
Traditional care	6 (4%)	7 (9%)	2 (7%)	1 (10%)	-	-
Community	5 (3%)	1 (1%)	-	-	-	-
Health centre	51 (31%)	14 (18%)	4 (14%)	3 (30%)	-	-
Hospital	81 (50%)	48 (63%)	21 (72%)	5 (50%)	4 (100%)	1 (100%)
Private	5 (3%)	6 (8%)	2 (7%)	1 (10%)	-	-

*In nine cases, we could not determine from the information provided in the open narrative if any care was sought

A total of 119/171 (68%) children ever visited a hospital, among whom 70% (n=83/119) were admitted. Amongst those admitted, caregivers specifically reported the child was given oxygen in 25% (n=21/83) of cases. Oxygen was more frequently reported amongst infants aged 1–11 months than those aged 12–59 months (32% vs 11%, p-value = 0.058), with no difference observed by sex (26% male vs 25% female). Pneumonia symptoms were recognised by caregivers less frequently in older children, but they were taken to hospital more frequently. Female children were taken to hospital more often than male children (74% vs 64%), but admitted less often than male children (68% vs 75%) – neither difference was statistically significant ( Table 4).

**Table 4.** Description of care-seeking behaviour, according to age and sex.

		Child’s sex *			Child’s age		
		Male (n=74)	Female (n=96)	p	1-11 months (n=124)	12-59 months (n=47)	p
Caregiver recognised pneumonia	No	33 (45%)	40 (42%)	0.639	44 (39%)	25 (53%)	0.087
	Yes	41 (55%)	56 (58%)		76 (61%)	22 (47%)	
Location of first seeking care	None	7 (9%)	7 (7%)	0.986 ^	14 (11%)	-	0.069 ^
	Hospital	35 (47%)	45 (47%)		58 (47%)	23 (49%)	
	Health centre	20 (27%)	31 (32%)		33 (27%)	18 (38%)	
	Community health worker	3 (4%)	2 (2%)		3 (2%)	2 (4%)	
	Private clinic	2 (3%)	3 (3%)		4 (3%)	1 (2%)	
	Traditional	3 (4%)	3 (3%)		6 (5%)	-	
	Unknown	4 (5%)	5 (5%)		6 (5%)	3 (6%)	
Location of death	Home	25 (34%)	39 (41%)	0.634 ^	53 (43%)	11 (23%)	0.028 ^
	Health centre or private clinic	4 (5%)	8 (8%)		7 (6%)	5 (11%)	
	Hospital	34 (46%)	38 (40%)		46 (37%)	27 (57%)	
	In transit	11 (15%)	11 (11%)		18 (15%)	4 (9%)	
Ever visited hospital	No	27 (36%)	25 (26%)	0.143	41 (33%)	11 (23%)	0.220
	Yes	47 (64%)	71 (74%)		83 (67%)	36 (78%)	
Ever admitted to hospital **	No	12 (26%)	23 (32%)	0.424	27 (32%)	9 (25%)	0.463
	Yes	35 (75%)	48 (68%)		56 (68%)	27 (75%)	
Received oxygen ***	No	26 (74%)	36 (75%)	0.845	38 (68%)	24 (89%)	0.058 ^
	Yes	9 (26%)	12 (25%)		18 (32%)	3 (11%)	

* In one case, the child’s sex is missing **Amongst those who ever attended hospital (N=118 for sex analysis; N=119 for age group analysis) ***Amongst those who were ever admitted to hospital (N=83) ^ Fisher’s exact test used.

**Figure 2.**
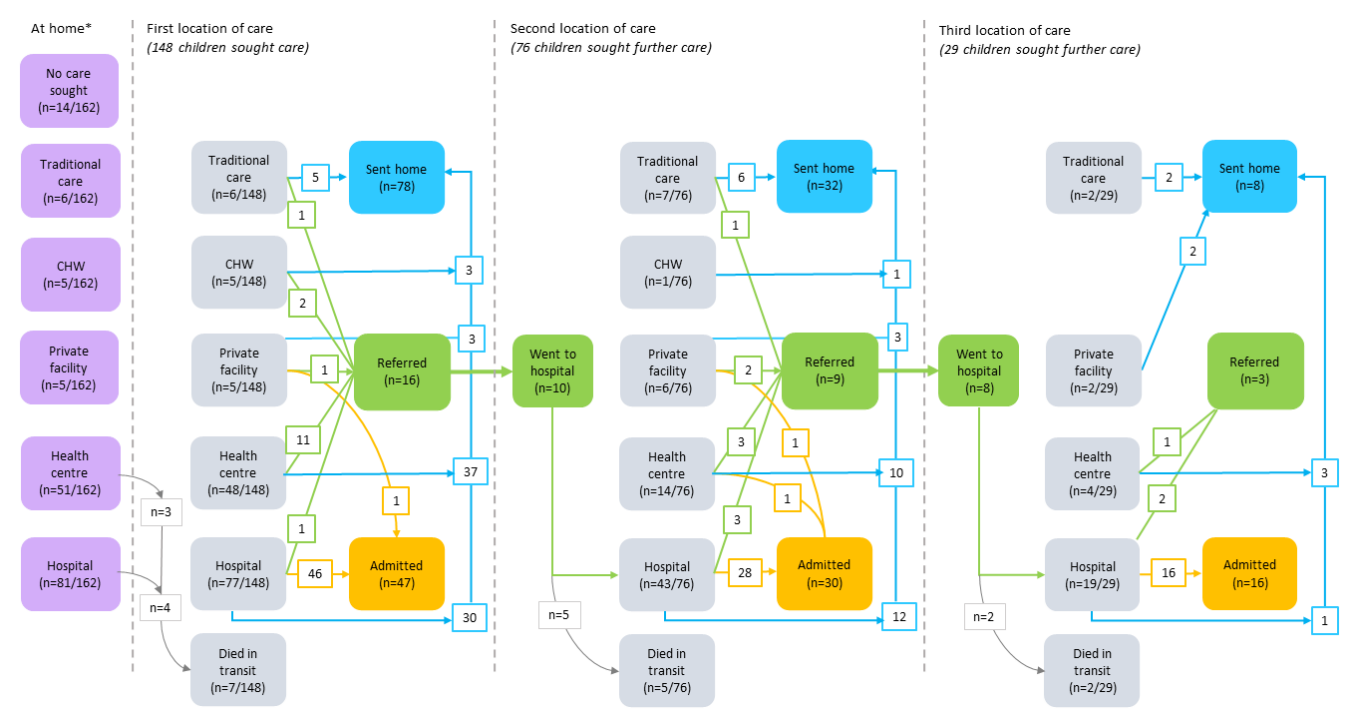
Summary of care-seeking according to the Pathways to Survival framework, for first, second and third location.

Half of the children died within a healthcare setting (43% hospital, 5% health centre and 2% private clinics). Of the 64 (37%) children who died at home, 42% (n=27/64) had visited a hospital and 17% (n=11/64) had been admitted; we were unable to extract time from discharge to death. The remaining 13% of children died in transit, either from their home to seek care, or during referral from primary to secondary care ( Figure 2). The majority of children aged 12–59 months died within a healthcare facility, while infants more often died at home (p-value = 0.028, Table 4).

### Qualitative analysis

***Wellness*.** Wellness includes prevent and protect indicators within households, with the sub-themes of: breastfeeding as a barometer of health; the role of a normal delivery; and sickly children. 

Breastfeeding was frequently used to express the overall health of the child. A child who was able to breastfeed, and a caregiver who was able to breastfeed their child from birth, were presented as being healthy. While the converse was presented both as a cause of illness in the child or a sign of severe illness.

*"the baby was ok, she was able to suck breastmilk"* (2 months, female)

*“He was not breastfeeding properly, because [I was] not producing milk. Then the child died”* (13 months, male)

Like the mother’s ability to breastfeed, the health of the child was linked to the mother having a ‘normal’ pregnancy and delivery. Narratives often started with the woman’s pregnancy, highlighting whether they had attended antenatal care or had an uncomplicated delivery. In several narratives, the respondents indicated that the child had been born prematurely. 

In a sub-group of narratives, caregivers described children who were repeatedly sick from recurrent and concurrent episodes of pneumonia, malaria, diarrhoea, and anaemia. In these cases, caregivers reported multiple episodes of care-seeking and described other comorbidities as explanations for their recurrent infections (e.g. prematurity, asthma, and congenital malformations). Misconceptions of asthma were apparent.

*“This baby has been ill on and off since she was born. We were not surprised because the baby was born with asthma, and another problem was that the baby was born with a large chest.”* (10 months, female.)

Conversely, there were narratives where respondents expressed surprise that their child was ill, referring to them having been vaccinated and growing strongly.

***Illness recognition*.** Illness recognition includes: pneumonia-specific signs and symptoms; overlap between pneumonia and asthma, and signs of severe illness.

Caregivers reported a range of clinical presentations for pneumonia, including; cough, difficulty breathing, noisy breathing, chest indrawing and fast breathing - in line with the IMCI chart booklet for diagnosing pneumonia. Caregivers differentiated between the severity of these different signs, with a cough often reported as the first sign of illness, which wasn’t necessarily treated, followed by the more serious sign of difficulty breathing. Fever was frequently reported, and was almost exclusively associated with malaria.

*"after some hours had passed the coughing started, and after a day or two passed the baby started having difficulties in breathing, so we went to the hospital."* (5 months, female.)

Several caregivers stated that their children were ‘born with asthma’ and that asthma triggered them to seek care, or their child died of a sudden asthma attack. However, it was often unclear from the narratives whether the child had asthma, pneumonia, or both. It should be noted that in the Chichewa transcripts, distinct terms are used for pneumonia ( *zibayo*) and asthma ( *mphumo*), and field staff reflected that they were not used interchangeably.

*"the child developed the problem of suffering from asthma - he had been admitted several times because of these attacks and during those times he was given medications like amoxicillin, Bactrim [cotrimoxazole], prednisone, Panadol and salbutamol."* (24 months, male.)

Danger signs that triggered the caregiver to consider the illness severe included fainting and convulsions. A commonly used term was ‘weakness’ of the child (e.g. “ *we just saw our child was weak”,* 12 months, female), and this was often provided as the trigger to seek care. This, and “dizzy eyes” may reflect the WHO general danger sign of lethargy. A child’s failure to breastfeed was also understood as being a sign of them being severely ill, and led to several caregivers stating that their children had died of starvation.

*“I believe that the child died of hunger because she was not feeding and coughing.”* (10 months, female.)

***Care-seeking decisions*.** Sub-themes within care-seeking decisions included: delaying seeking care until the illness was severe; and the plurality of care. In several cases the respondent stated that they recognised the child was unwell, but did not seek care immediately. Rather they waited until a more severe sign of illness was seen (e.g. weakness, failing to feed or difficulty breathing).

In Malawi, traditional and western medicine exist in parallel. This plurality in healthcare systems was apparent in several narratives with caregivers reporting attending both types of care for the same episode of illness.

*"the baby kept on crying and could hardly be stopped. We thought it could be evil spirits… therefore traditional medicine was given but there was no change, so we decided to visit the health centre."* (4 months, male.)

The sequential access of different types of care, was attributed to the failure of the care sought either to cure the child or a perception that the care was not sufficient, or on the advice of neighbours and relatives:

*"the child was given some panadols […] we therefore resorted to traditional drugs."* (6 months, female.)

***Referral procedures*.** Referral procedures, covering both healthcare worker’s recommendation to seek further care and the process of getting to further care, included the sub-themes of: community advice; transportation barriers; and advice to wait and see. Advice to initially seek care and further care, was received from different community sources, namely relatives, traditional birth attendants and community healthcare workers. It should be noted respondents reported that community members recommended further care be sought both through formal and informal providers.

*"when we reached home we were advised to proceed to [the hospital] and indeed we started off for the hospital."* (10 months, female.)

Following community and healthcare provider referrals, challenges in getting to the facility due to transportation issues were reported. These barriers included lack of ambulance access, issues in finding the funds to arrange private transport and in the example given, the inappropriate use of an ambulance.

*"the ambulance that carried us also took some nurses who had different errands, and we stopped at the market. By the time we reached [the hospital] the child was pronounced dead."* (10 months, male.)

There were several stories of children taken to health centres, given treatment and sent home with the advice to return if the child did not improve –in line with WHO IMCI protocols for children without severe illness.

*"the doctor said that if we see no signs of any change, we should go to bigger hospital… there was no change and indeed we went to [the hospital].”* (16 months, female.)

***Quality of care*.** Several quality of care challenges were reported during the open narratives, relating both to pneumonia specific issues, and wider health system challenges. Sub-themes include challenges in oxygen provision, barriers to blood provision, inappropriate discharge, missed opportunities to treat, and delays in care provision.

Oxygen was reported by many caregivers as being given at the time of the child’s death, with many children dying shortly after being given oxygen. There were three specific narratives where caregivers reported the power going out while receiving oxygen, resulting in the child’s death.

*"He was put on oxygen for 50 minutes… shortly after the electricity at the hospital went out and the doctors removed the child from the oxygen machine. After 20 minutes the electricity was on again and the doctors came to take the child and put them on the oxygen machine. […] unfortunately they found that the child was already dead."* (7 months, male.)

Many caregivers also reported that their child was prescribed a blood transfusion, but issues inserting intravenous lines and the lack of available blood meant they didn’t receive it. A major gap in quality of care reported from caregivers was that their child was discharged from hospital while they were still unwell:

*"Although she was discharged the child was not okay…very shortly after, it’s when the child died here at home, the same day she was discharged from hospital."* (8 months, female.)

Caregivers reported several situations of missed opportunities for diagnosis and treatment, including lacking medications and failing to diagnose the child with any illness during their examination. In these cases, children were sent home with no diagnosis, treatment or referral, leading to delays in care provision.

*"we took the baby to [the clinic] and there was no medication at the facility. We went back home and the coughing continued… come the next day, the baby did not survive."* (2 months, male.)

Within facilities, some reported delays in the child being seen by a healthcare provider, and in one such case the caregiver attributed her child’s death to this delay:

*"I blame the negligence of medical personnel at the hospital for delaying admission, and the time they took to start assisting the baby.*" (8 months, male.)

## Discussion

In this mixed-methods analysis, we found high levels of caregiver recognition of illness, the majority of children who died of suspected pneumonia had accessed formal healthcare, and multiple interactions with healthcare were common. The narratives highlighted several missed opportunities for earlier intervention, including challenges in pneumonia recognition and diagnosis, getting to healthcare, attending referrals and issues in quality of care. 

In absolute numbers, there were more deaths in girls than boys (96 versus 74). Hospital data from the same setting reported more male than female admissions, however, and being female was a predictor of inpatient pneumonia mortality ^20^. This echoes evidence from LMICs that females have a higher pneumonia mortality risk ^2^. One hypothesis for this is differential gendered care-seeking ^21^, but we found no clear supporting evidence; rather, female children were taken to hospital more often (74% versus 64%, p-value = 0.143). A more in-depth investigation into the biological and cultural reasons for these sex differences is still needed.

We observed interesting distinctions in care between infants (1–11 months) and older children (12–59 months). Pneumonia symptoms were less frequently recognised by caregivers in older children, but they were more likely to be admitted and die within a hospital setting. One explanation could be due to infants having more compliant chest walls, chest indrawing as clinical sign is less specific to pneumonia in this group ^22^; alongside the inability of younger children to express themselves, this may result in poorer case management. Further investigation of how illness recognition by caregivers and healthcare providers changes with age could provide important insights for improving care.

The most common location to first seek care was the hospital. Further, of those who first attended a health centre and were referred to hospital, 63% (n=10/16) adhered to this recommendation. A study from central Malawi found caregivers accepted referrals from health centres to hospitals in 58% of critical cases but in just 4% of severe cases ^23^. This suggests caregivers recognise the seriousness of their child’s condition. However, the short interval between illness recognition and death (median three days), could mean early and non-severe signs of illness which can be treated at the community level were missed; a notion supported by research from Mozambique ^24^. Caregivers rarely reported seeking care at the community-level despite Malawi’s mature iCCM programme. A recent study reported caregiver preferences for non-community health workers and that issues in service coverage and quality of care remain ^25^, possibly explaining why community care was bypassed. We did not ask caregivers about their decision making processes and motivations around care-seeking, which would give more insight.

Multiple deficiencies in quality of care, as outlined in WHO Quality of Care Framework ^26^, were described. Namely the lack of drugs, availability of staff, functionality of referral systems and respectful communication - leading to multiple delays in care. Indeed, data from this setting shows that children referred from health centres have an increased odds of in-patient mortality, compared to those coming directly from home (aOR: 1.90; 95% CI: 1.25-2.89) ^27^. Similar to caregivers bypassing CHWs, we also found that caregivers bypassed health centres despite often being closer and able to provide treatment for pneumonia. Efforts to strengthen emergency case management, including effective triage, alongside routine IMCI provision in primary care may allow for quicker access to treatment in critical cases. However, sustainable strategies to improve healthcare worker performance need to go beyond basic training ^28^, and be supported with access to essential resources. 

A key quality of care issue for pneumonia is access to reliable oxygen, and several caregivers reported their child dying while receiving oxygen, often after a short treatment period. Group discussions with caregivers in Malawi found negative community perceptions of oxygen, with oxygen equated with child deaths ^29^. While we did not see this conception reflected, it may have contributed to delayed oxygen treatment. Pulse oximetry was implemented in all health centres and hospitals in the district as part of a concurrent research project, with 89% of routinely documented clinical pneumonia patients having an oxygen saturation ^30^. Despite access to pulse oximetry, children in need of oxygen were still not effectively treated. Unreliable power supplies have been repeatedly reported as a barrier to effective oxygen delivery in LMICs ^31^, and we observed cases where this contributed to child deaths. However, oxygen provision with cylinders and back-up power or solar-powered oxygen systems should allow for solutions to this issue ^32^. It is important that investment in oxygen systems consider wider targeting of pulse oximetry screening, on-going mentorship and supervision, equipment maintenance, and locally appropriate power solutions to maximise impact ^33^.

Our study had three key limitations. Firstly, we only considered caregiver perspectives of the circumstances surrounding their child’s death and were unable to verify clinical details (e.g. oxygen). Secondly, while the narratives were purposefully collected in an unstructured manner, we were unable to distinguish if the absence of reporting means it didn’t happen, was not stated by the caregiver, or not documented by the fieldworkers. As caregivers narrated the story of the child’s death from their own perspective, it may be subject to both recall and social desirability biases. Thirdly, not all of the deaths defined as suspected pneumonia are necessarily due to pneumonia and we may have missed pneumonia deaths. There were clear overlaps in the narratives between pneumonia, malaria and asthma. Given most deaths were assigned as ‘pneumonia’ by InterVA, which is designed to give population level cause-specific mortality fractions, pneumonia-specific conclusions should be interpreted with caution.

Paediatric pneumonia is a complex condition, requiring a whole systems perspective, covering protect and prevent interventions, through to quality delivery of healthcare services. We observed shortfalls across this spectrum, but particularly around missed opportunities to better manage cases which presented to healthcare. Most caregivers brought their child to healthcare, and many sought care multiple times from different healthcare providers, suggesting sub-optimal pneumonia case management. Improved implementation of existing iCCM and IMCI protocols with feedback and accountability systems are needed, in conjunction with further research on optimised diagnosis, treatment and referral approaches in primary care, to reduce paediatric pneumonia deaths.

## Data availability

### Underlying data

Harvard Dataverse: Replication Data for: "Care-seeking patterns amongst suspected paediatric pneumonia deaths in rural Malawi: a mixed method study"

https://doi.org/10.7910/DVN/66YIME ^34^

This project contains the following underlying data:

- VA_OH_CleanRepositoryData.xls (Fully anonymised quantitative data used in analysis.)

Data are available under the terms of the Creative Commons Zero "No rights reserved" data waiver (CC0 1.0 Public domain dedication).

The open narratives cannot be sufficiently anonymised, as they contain detailed accounts of individual death events with locations, dates, demographic and clinical details. These are therefore not available in an open repository. For researchers wishing to duplicate or re-analyse these data for data for research purposes only, contact Dr Carina King ( carina.king@ki.se) or Prof. Neil French ( N.French@liverpool.ac.uk), to discuss the signing of a data sharing agreement, following review by the National Health Sciences Research Ethics Committee in Malawi (contact: infor@ncst.mw).
