# Supplementary material for: Care-seeking patterns amongst suspected paediatric pneumonia deaths in rural Malawi
Source: Gates Open Res. 2021 May 6;4:178. Originally published 2020 Dec 4. [Version 2] doi: 10.12688/gatesopenres.13208.2 (PMC7835598; doi:10.12688/gatesopenres.13208.2)
Supplement: Peer review history

# Peer review: Reviewer response for version 1

**DOI:** 10.21956/gatesopenres.14424.r30179

**Published:** 2021-01-22

**Authors:** Christopher J. Gill

**Affiliations:** 1 Department of Global Health, Boston University School of Public Health, Boston, MA, USA

The authors present a useful mixed methods analysis of verbal autopsy data collected In Malawi among children who died of suspected pneumonia. Overall, the methodology was rigorous and logical and I only had a few queries around areas that were not quite clear. The conclusions drawn were persuasive and consistent with similar analyses regarding the role of delays in care seeking. 

 Minor comments/queries:

1. I wasn’t clear about why a free text narrative was not done in all verbal autopsies. The authors mention that there were theoretical concerns about the impact of the interview (leading to the narrative) on data quality and rapport with the person being interviewed.  However, the free text narrative typically occurs at the end of the VA interview, so I’m not clear how this could then affect the rest of the interview, which should have occurred already. I don’t see this as a source of bias, since it was driven by randomization. But it did reduce the information available to the team in half of the children. Please explain this further. 
2. Table 2 refers to ‘reported signs’ as part of the pneumonia classification process. However it is not clear what this means. Earlier in the methods the authors mention use of several of the VA questions as being part of the classification strategy, but the terminology ‘signs’ is not used there. Please clarify. 
3. Minor issue, explain what ‘attended hospital’ means. 
4. I’m curious about the much higher proportion of home deaths among infants. Is that because there is a tendency against care seeking? Is it that signs of Pna are often subtle and therefore easily missed in infants? Is it that progression to death is more rapid? Or possibly a combination of these factors? Please comment. 

Is the work clearly and accurately presented and does it cite the current literature?

Yes

If applicable, is the statistical analysis and its interpretation appropriate?

Yes

Are all the source data underlying the results available to ensure full reproducibility?

Yes

Is the study design appropriate and is the work technically sound?

Yes

Are the conclusions drawn adequately supported by the results?

Yes

Are sufficient details of methods and analysis provided to allow replication by others?

Yes

Reviewer Expertise:

Pediatric pneumonia and respiratory infections

I confirm that I have read this submission and believe that I have an appropriate level of expertise to confirm that it is of an acceptable scientific standard.

# Response

**Published:** 2021-03-01

**Authors:** Carina King

**Affiliations:** Karolinska Institutet, UK

1. I wasn’t clear about why a free text narrative was not done in all verbal autopsies. The authors mention that there were theoretical concerns about the impact of the interview (leading to the narrative) on data quality and rapport with the person being interviewed.  However, the free text narrative typically occurs at the end of the VA interview, so I’m not clear how this could then affect the rest of the interview, which should have occurred already. I don’t see this as a source of bias, since it was driven by randomization. But it did reduce the information available to the team in half of the children. Please explain this further. 

***Response:** The randomisation to have an open narrative or not was done as an opportunistic embedded study to explore the role of narratives in the process of verbal autopsies. While the standard WHO VA tool includes a narrative, this is optional and often not analysed when cause of death is assigned using automated methods. In this study we had decided to conduct the open narrative at the start of the VA interview. We have published a paper which reports our findings on this separate research question, and reference this in the text.*

**

 2. Table 2 refers to ‘reported signs’ as part of the pneumonia classification process. However, it is not clear what this means. Earlier in the methods the authors mention use of several of the VA questions as being part of the classification strategy, but the terminology ‘signs’ is not used there. Please clarify. 

***Response: **Reported signs in Table 2 refers to the caregiver responding “yes” to the child having a cough and difficulty breathing in the two weeks prior to death. We have updated the label in the table to use the same language as the methods.*

 3. Minor issue, explain what ‘attended hospital’ means. 

***Response:*** *This means they went to hospital, but were not necessarily admitted. We have rephrased to “visited hospital” and hope this is clearer language.*

**

 4. I’m curious about the much higher proportion of home deaths among infants. Is that because there is a tendency against care seeking? Is it that signs of Pna are often subtle and therefore easily missed in infants? Is it that progression to death is more rapid? Or possibly a combination of these factors? Please comment.

***Response: **This is a really important point, and one which we can only make general comments and reflections on. Rates of care-seeking were similar between infants and older children, but we saw higher rates of hospitalisation and oxygen treatment in the older age group. There are several reasons this may be, firstly that signs of severe respiratory distress were more easily recognised in older children (aided by children being able to say how they are feeling). Secondly, when pulse oximetry is available, it is generally easier to get measurements on older children. It may be that hypoxaemia in older children was identified more often. This is especially important as we’ve seen in this setting before that a lot of hypoxemic children are missed according to current IMCI guidelines (e.g. McCollum et al., 2016). Finally, there may be cultural perceptions around care and illness in different age groups that we have not accounted for.* *Further work is definitely warranted to understand motivations, decision making processes and bottlenecks in care-seeking pathways, across a range of contexts. In particular, a study which explores how different clinical signs of pneumonia are recorded across age groups and whether they are seen are equally serious by both caregivers and healthcare workers would be valuable.*

# Peer review: Reviewer response for version 1

**DOI:** 10.21956/gatesopenres.14424.r30102

**Published:** 2020-12-21

**Authors:** Prudence Hamade

**Affiliations:** 1 Malaria Consortium, London, UK

This is a very well written and very important article with comprehensive data from a study using verbal autopsy approach described by WHO. The limitations of this approach are well described. 

 I think a table of the components and indicators of the Pathway to Survival would have been good to include so it could be compared to the components chosen for this analysis especially as although the data was collected by open narrative they were coded by predetermined themes'

 There is a strong description in this paper of what care people sought but little information of why they sought care in those places. The diagram explaining the care seeking behaviour was very interesting but is limited in its explanation of why people sought care where they did and what were the actual pathways chosen. For example if a person sought care from a traditional healer in the first instance where did they go to seek care in the second instance. The belief that medicine is magic and cure will be immediate in my experience leads to going from one care provider to another with prescription of multiple medications and then decision by the care giver of those they can afford to buy and administer causing possible toxicity in the child as well as under dosing of the appropriate therapy.

 In the first question I think the recognition of illness should not of excluded witchcraft or curses as this is an important issue around care seeking especially as some people sought care from traditional healers.

 No mention in the accounts of seeking care from shops and pharmacies which are a common source of care seeking ( cheap and available at all time of day).

 The low number of people who sought care from community health workers is interesting but the reason for this is not explained.

 It would be interesting to know how many caregivers of children sought care from community health workers whose children did not die  to compare with the health seeking behavior of children with symptoms of pneumonia who did die.

 The data does not clearly show what percentage of the children had risk factors associated with death although some of these are described such as prematurity failure to thrive etc. 

 The attribution of asthma is also very interesting. Asthma is not frequently diagnosed or treated in LMICs also non infectious causes of difficulty breathing like bronchiolitis which if not treated correctly can lead to death.

 Training of health care workers especially at hospital level in use of supportive measure like use of pulse oximetry, oxygen and bronchodilators needs to be improved as well as what was not fully emphasized is triage at the health facility level to make sure that severely ill children receive urgent care when they arrive at the hospital.

Is the work clearly and accurately presented and does it cite the current literature?

Yes

If applicable, is the statistical analysis and its interpretation appropriate?

Yes

Are all the source data underlying the results available to ensure full reproducibility?

Yes

Is the study design appropriate and is the work technically sound?

Yes

Are the conclusions drawn adequately supported by the results?

Yes

Are sufficient details of methods and analysis provided to allow replication by others?

Partly

Reviewer Expertise:

I am a retired Community Paediatrician. I have worked for 10 years in the field with MSF and 11 years with Maria Consortium mainly in the field of infectious disease and child health

I confirm that I have read this submission and believe that I have an appropriate level of expertise to confirm that it is of an acceptable scientific standard.

# Response

**Published:** 2021-03-01

**Authors:** Carina King

**Affiliations:** Karolinska Institutet, UK

I think a table of the components and indicators of the Pathway to Survival would have been good to include so it could be compared to the components chosen for this analysis especially as although the data was collected by open narrative they were coded by predetermined themes.

***Response:*** *We have now added a sentence to the methods to highlight the components of the framework which we did not include in the data extraction. These were mostly around contextual wellbeing factors, rather than the indicators for care-seeking and actions taken.*

 There is a strong description in this paper of what care people sought but little information of why they sought care in those places. The diagram explaining the care seeking behaviour was very interesting but is limited in its explanation of why people sought care where they did and what were the actual pathways chosen. For example if a person sought care from a traditional healer in the first instance where did they go to seek care in the second instance. The belief that medicine is magic and cure will be immediate, in my experience leads to going from one care provider to another with prescription of multiple medications and then decision by the care giver of those they can afford to buy and administer, causing possible toxicity in the child as well as under dosing of the appropriate therapy.

***Response: **This is an excellent point, and the access of different care for different motivations happening concurrently has been described in Malawi previously. Reasons for this have been that some causes of illness need to be treated with traditional medicine, or when treatments do not seem to be working other forms of care are sought. However, in this study we did not probe around motivations for the decisions made, and reasoning or motivations for care-seeking actions was not something which emerged from the narratives. We would love to see more in-depth research into this topic area however, to ensure health systems are responsive to communities.*

 In the first question I think the recognition of illness should not of excluded witchcraft or curses as this is an important issue around care seeking especially as some people sought care from traditional healers.

***Response: **In the caregiver recognition of illness, we accepted any plausible clinical symptom (e.g. fever, cough) and phrases such as “the child was sick”. These non-specific terms may cover illnesses perceived to be either due to medical causes or witchcraft, and we did not attempt to differentiate. However, we did not actively classify text such as “the child was cursed” as a recognition of illness. Checking the data, there was one narrative where the caregiver reported the child had been cursed (“njazi”) and was coded as no illness recognised as no further signs or symptoms was given.*

 No mention in the accounts of seeking care from shops and pharmacies which are a common source of care seeking (cheap and available at all time of day).

***Response: **This is a great observation, and to confirm care-seeking from pharmacy shops was not explicitly mentioned in the narratives. In the context, care is provided free at government health facilities, and at the time of the study, private facilities and pharmacies within rural settings were relatively uncommon. We have added a statement to make this explicit in the text.*

 The low number of people who sought care from community health workers is interesting but the reason for this is not explained.

***Response: **As in the response above we did not probe around motivations for care-seeking decisions. However, we agree that in this context with well-established community care it would be valuable to understand when and why caregivers would seek care in the community versus travelling to health centres or the district hospital, which were common.*

 It would be interesting to know how many caregivers of children sought care from community health workers whose children did not die to compare with the health seeking behavior of children with symptoms of pneumonia who did die.

***Response:** This is a limitation of the data we had available. We did not actively follow-up children who had pneumonia and survived, so do not have a corresponding dataset for surviving children to compare. This analysis however would be valuable to try and identify if care-seeking pathways diverge at any point, and therefore identify the key bottlenecks.*

 The data does not clearly show what percentage of the children had risk factors associated with death although some of these are described such as prematurity failure to thrive etc. 

***Response: **We have now added this in the results.*

 The attribution of asthma is also very interesting. Asthma is not frequently diagnosed or treated in LMICs also non-infectious causes of difficulty breathing like bronchiolitis which if not treated correctly can lead to death.

***Response: **Yes, this was a really interesting finding and through more informal discussions seems that more chronic difficulties in breathing are talked about as asthma but more acute issues are pneumonia. This does raise questions on how we can improve both the diagnosis and management of asthma, when there has been a lot of focus on diagnosing and treating infections within primary care settings.*

 Training of health care workers especially at hospital level in use of supportive measure like use of pulse oximetry, oxygen and bronchodilators needs to be improved as well as what was not fully emphasized is triage at the health facility level to make sure that severely ill children receive urgent care when they arrive at the hospital.

***Response:** We fully agree with this, and have amended the text in the manuscript to make this point about improved triage.*
